# Supplementary material for: Topological–chiral magnetic interactions driven by emergent orbital magnetism
Source: Nat Commun. 2020 Jan 24;11:511. doi: 10.1038/s41467-019-14030-3 (PMC6981145; doi:10.1038/s41467-019-14030-3)
Supplement: Supplementary file 1 — Supplementary Information [file 41467_2019_14030_MOESM1_ESM.pdf]

Topological-chiral magnetic interactions driven by emergent orbital magnetism

Grytsiuk et al.

Supplementary Figures

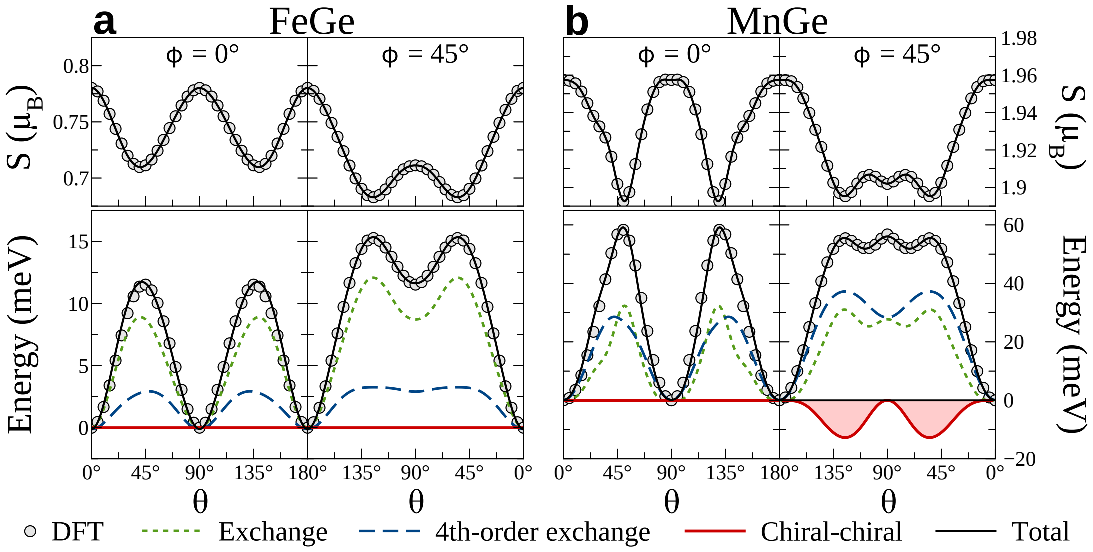

Supplementary Figure 1 | Role of variation of spin-moment length. Change of the spin-moment length S (top) and the different contributions to the total energy (bottom) as a function of the angle θ for fixed $\phi = 0°$ and 45° in **a**, FeGe and **b**, MnGe. Circles mark the first-principles results, and lines correspond to the fitted angular dependencies. The shapes of the exchange interactions in the effective spin model include the changes of the spin-moment length.

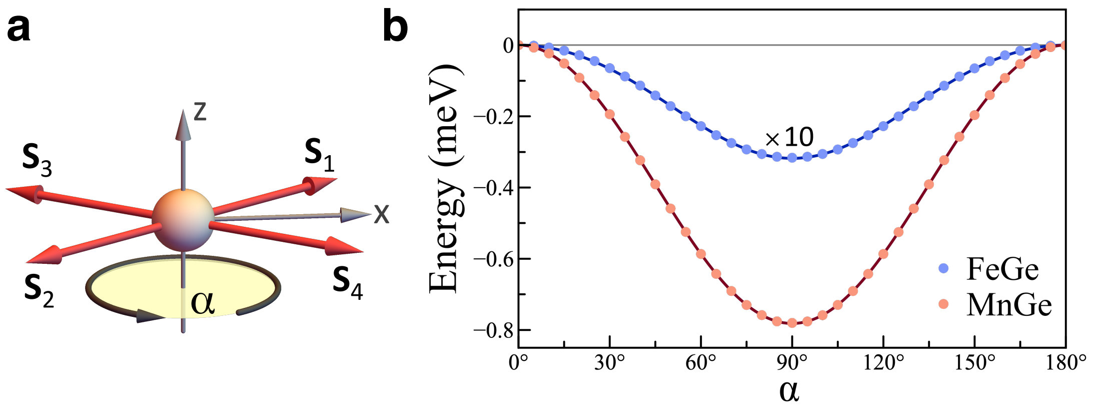

Supplementary Figure 2 | Magnetocrystalline anisotropy in non-collinear B20 magnets. (a) Considered coplanar magnetic structure that is rotated by the angle α around the z axis. (b) Spin-orbit energy in FeGe (multiplied by 10) and MnGe as a function of α, where the solid line is a fit of the model in Supplementary Eq. (37) to the first-principles data points.

Supplementary Tables

Supplementary Table 1 | Effective fitting parameters for the exchange interactions used in the main text and Supplementary Notes 5 and 6, where a constant length of the spin magnetic moment is assumed.

Calculations	without SOI			with SOI				
Parameters	K (meV)	$\varkappa^{\mathrm{CC}}$ (meV)	κ^{TO} (μ_{B})	D (meV)	$\varkappa^{\mathrm{SC}}$ (meVμ_{B}^{-1})	$\varkappa^{\mathrm{SC}}\kappa^{\mathrm{TO}}$ (meV)	A_{uniax} (meV)	A_{cubic} (meV)
FeGe	11.77	0.30	−0.02	−1.85	−12.40	0.21	−0.03	1.7×10^{-3}
MnGe	58.24	21.76	0.13	1.03	−25.31	−3.17	−0.78	-0.5×10^{-3}

Supplementary Table 2 | Effective fitting parameters for the exchange interactions without spin-orbit coupling, where the length of the spin magnetic moment changes according to Supplementary Eq. (26).

Parameters	J (meV)	K (meV)	K' (meV)	$\varkappa^{\mathrm{CC}}$ (meV)	κ^{TO}(μ_{B})
FeGe	7.18	2.17	0.88	0.00	−0.02
MnGe	22.27	5.64	1.10	10.22	0.14

Supplementary Notes

Supplementary Note 1 | Derivation of the topological-chiral magnetic interactions from electronic structure theory

In this Supplementary Note we derive the expressions for the topological-chiral interactions by a systematic expansion of the total energy of a many-electron system with respect to simultaneous infinitesimal rotations of the magnetic moments. We consider a solid that is described by density functional theory. This is not a necessary condition. Our derivations can be extended to strongly correlated electron systems following the work of Ref. [1]. By rotating a set of magnetic moments within this solid, their mutual interaction can be obtained from the change of the total energy. Since these rotations at different sites are inhomogeneities on top of the otherwise unperturbed solid, a formalism based on Green functions corresponding to the Kohn-Sham Hamiltonian of the solid is ideal.

To extract the interaction parameters, we invoke a scattering formalism realised by the Korringa-Kohn-Rostoker (KKR) Green-function formalism, where the Green function, G, describes the propagation of the electron states from site i to site j and the scattering strength at those sites is described by the single-site scattering matrix t. Within this approach, the infinitesimal rotation of the magnetic moments is sufficient to extract the magnetic exchange interactions (MEI) as usually done to obtain the bilinear Heisenberg-type MEI [2–4].

In this case, the total energy can be perfectly approximated by the band energy and the energy difference due to rotations is given as

$$\delta E = -\frac{1}{\pi}\mathrm{Im}\int_{-\infty}^{\epsilon_F} \mathrm{d}\epsilon\ \mathrm{Tr}\ln\left[1 - G(\epsilon)\delta t(\epsilon)\right], \tag{1}$$

where the matrices G and δt are respectively the Green function of the electronic system prior to the perturbation and the change of the single-site scattering matrix after rotating the magnetic moments. In the KKR formulation, the Green function and the scattering matrix are represented in the angular momentum $L = (l, m)$ and spin representation, $L \oplus s$. The trace Tr is taken over the sites i, orbital momentum $L = (l, m)$, and spin indices.

The Green function that connects the atomic sites i and j can be partitioned into two matrices:

$$G_{ij} = A_{ij}\,\sigma_0 + \mathbf{B}_{ij}\cdot\boldsymbol{\sigma}\,, \tag{2}$$

where A_{ij} is the non-magnetic part of the Green function diagonal in spin space, while $\mathbf{B}_{ij}$ contains simultaneously the magnetic part and a contribution originating from the spin-orbit interaction (SOI). $\boldsymbol{\sigma}$ is the vector of Pauli matrices and σ_0 is the identity matrix. We note that if inversion symmetry is not broken, A_{ij} is symmetric with respect to site exchange and likewise for $\mathbf{B}_{ij}$ if

SOI is not present. Similarly to the Green function, the single-site t-matrix can be written in terms of non-magnetic and magnetic parts:

$$t_i(\epsilon) = \frac{1}{2}(t_i^{\uparrow}(\epsilon) + t_i^{\downarrow}(\epsilon))\,\sigma_0 + \frac{1}{2}(t_i^{\uparrow}(\epsilon) - t_i^{\downarrow}(\epsilon))\,\mathbf{S}_i \cdot \boldsymbol{\sigma}, \tag{3}$$

where $t^{\uparrow}$ and $t^{\downarrow}$ are the majority- and minority-spin t-matrices as obtained in the local-spin frame of reference of each atom. $\mathbf{S}_i$, as defined in the main text, is the unit vector of the spin magnetic moment at site i. Changing the orientation of the magnetic moment of a given atom and adopting the rigid spin approximation implies a corresponding change in the spin-dependent t-matrix

$$\delta t_i(\epsilon) = t_i^{\mathrm{s}}(\epsilon)\,\delta\mathbf{S}_i \cdot \boldsymbol{\sigma}\,, \tag{4}$$

in which $t^{\mathrm{s}} = \frac{1}{2}(t_i^{\uparrow}(\epsilon) - t_i^{\downarrow}(\epsilon))$ is the magnetic part of the total t-matrix if SOI is neglected.

Considering infinitesimal rotations, we proceed to a Taylor expansion of the logarithm in Supplementary Eq. (1) and extract systematically high-order terms ordered according to powers of δt . Terms with odd powers of δt cancel since they are not compatible with the time-reversal symmetry requirement of the total energy. For instance, the standard second order term

$$\delta E_{2\text{-spin}} = -\frac{1}{2\pi}\mathrm{Im\ Tr}\int_{-\infty}^{\epsilon_F} \mathrm{d}\epsilon \sum_{ij} G_{ij}(\epsilon)\delta t_j(\epsilon) G_{ji}(\epsilon)\delta t_i(\epsilon) \tag{5}$$

$$= -\sum_{ij}\left[J_{ij}^{\mathrm{iso}}\delta\mathbf{S}_i\cdot\delta\mathbf{S}_j + \delta\mathbf{S}_i\cdot\underline{\underline{J}}_{ij}^{\mathrm{ani}}\cdot\delta\mathbf{S}_j + \mathbf{D}_{ij}\cdot\left(\delta\mathbf{S}_i\times\delta\mathbf{S}_j\right)\right] \tag{6}$$

leads to the well-known interaction energy between two magnetic moments from which the bilinear magnetic exchange interactions can be extracted: the isotropic MEI, J^{iso}, the two-site anisotropy tensor of MEI, $\underline{\underline{J}}^{\mathrm{ani}}$, and the Dzyaloshinskii-Moriya interaction (DMI) with the DM vector [5, 6], $\mathbf{D}$. $\underline{\underline{J}}^{\mathrm{ani}} \in \mathbb{R}^{3\times 3}$ represents a (trace-less symmetric) matrix in the space of spin-moment orientation. While these interactions are well-known, it is interesting to express the magnetic exchange interactions in terms of the Green functions as decomposed in Supplementary Eq. (2):

$$J_{ij}^{\mathrm{iso}} = \frac{1}{\pi}\mathrm{Im\ Tr}\int_{-\infty}^{\epsilon_F}\mathrm{d}\epsilon\left\{A_{ij}(\epsilon)t_j^s(\epsilon)A_{ji}(\epsilon)t_i^s(\epsilon) + \left[\mathbf{B}_{ij}(\epsilon)t_j^s(\epsilon)\right]\cdot\left[\mathbf{B}_{ji}(\epsilon)t_i^s(\epsilon)\right]\right\} \tag{7}$$

and

$$\mathbf{D}_{ij} = \frac{1}{\pi}\mathrm{Re\ Tr}\int_{-\infty}^{\epsilon_F}\mathrm{d}\epsilon\left\{A_{ij}(\epsilon)t_j^s(\epsilon)\mathbf{B}_{ji}(\epsilon)t_i^s(\epsilon) - \mathbf{B}_{ij}(\epsilon)t_j^s(\epsilon)A_{ji}(\epsilon)t_i^s(\epsilon)\right\}. \tag{8}$$

Here one sees explicitly, how the spin-orbit interaction field encoded in $\mathbf{B}$ affects the different MEI. As expected, J has a contribution independent of magnetism and SOI, as well as a contribution which depends on both. The latter contribution is at least quadratic with respect to the SOI, which occurs in form of an inner product of the different $\mathbf{B}$, and thus the interaction is rotationally invariant in spin-space. $\mathbf{D}$, in contrast, is linear in $\mathbf{B}$ and thus linear in the SOI, and the direction of $\mathbf{D}$ is determined by the vector properties of $\mathbf{B}$ whose details are determined by the underlying lattice as well as the two atomic sites i and j between which the interaction mediates. The latter defines the symmetry properties required for a non-vanishing DMI. For lattices with inversion symmetry, we naturally recover that the DMI vanishes because of the symmetry of A and $\mathbf{B}$ with respect to site exchange.

The fourth-order term in the Taylor expansion of the energy,

$$\delta E_{4\text{-spin}} = -\frac{1}{4\pi}\mathrm{Im\ Tr}\int_{-\infty}^{\epsilon_F}\mathrm{d}\epsilon\sum_{ijkl} G_{ij}(\epsilon)\delta t_j(\epsilon)G_{ji}(\epsilon)\delta t_k(\epsilon)G_{kl}(\epsilon)\delta t_l(\epsilon)G_{li}(\epsilon)\delta t_i(\epsilon)\,, \tag{9}$$

involves plaquettes of four magnetic moments. Analogous to the 2-spin interaction, $E_{4\text{-spin}}$ gives rise to the conventional isotropic 4-spin interactions [7] proportional to $(\mathbf{S}_i\cdot\mathbf{S}_j)(\mathbf{S}_k\cdot\mathbf{S}_l)$ including quadratic and fourth order contributions of the SOI, as well as to terms linear in the SOI. The latter are the recently derived [8] and postulated [9] 4-spin vector-chiral interactions proportional to $(\mathbf{S}_i\cdot\mathbf{S}_j)(\mathbf{S}_k\times\mathbf{S}_l)$ and the newly put forward spin-chiral contributions,

$$\delta E_{ijkl}^{\mathrm{SC}} = -\left(\boldsymbol{\mathcal{K}}_{ijk\bar{l}}\cdot\delta\mathbf{S}_l\right)\left[\delta\mathbf{S}_i\cdot\left(\delta\mathbf{S}_j\times\delta\mathbf{S}_k\right)\right] - \left(\boldsymbol{\mathcal{K}}_{i\bar{j}kl}\cdot\delta\mathbf{S}_j\right)\left[\delta\mathbf{S}_i\cdot\left(\delta\mathbf{S}_k\times\delta\mathbf{S}_l\right)\right]\,, \tag{10}$$

to the topological-chiral interaction, E^{SC}, introduced in the main text involving the scalar spin-chirality $\chi_{ijk} = \mathbf{S}_i\cdot(\mathbf{S}_j\times\mathbf{S}_k)$. Without loss of generality, we have chosen within our derivation oriented plaquettes with a counterclockwise circulation of the site indices $(ijkl)$. Similarly to the DMI, $\boldsymbol{\mathcal{K}}_{ijkl}$ is a vector quantity, which is linear in $\mathbf{B}$ connecting pairs of atomic sites within the plaquette of sites $(ijkl)$. This indicates that the direction of $\boldsymbol{\mathcal{K}}$ should be determined by symmetry rules related to those of the DMI. The product of the four Green-function elements in Supplementary Eq. (9) and the requested linearity in $\mathbf{B}$ leads finally to

four terms of the type $AAA\mathbf{B}$, where $\mathbf{B}$ can be placed at four different positions relative to A. Since $\mathbf{B}_{\cdot j}$ can form inner products with the unit vector $\mathbf{S}_j$ and vertex j can connect to vertices (ij), (jk) as well as vertex l can connect to the vertices (kl), (li), we can group finally the four contributions to the vector interaction parameter $\boldsymbol{\mathcal{K}}_{ijkl}$ in two groups as following

$$\boldsymbol{\mathcal{K}}_{ijk\bar{l}} = \frac{1}{2\pi}\mathrm{Re}\ \mathrm{Tr}\int_{-\infty}^{\epsilon_F}\mathrm{d}\epsilon\,\{A_{ij}(\epsilon)t_j^s(\epsilon)A_{jk}(\epsilon)t_k^s(\epsilon)A_{kl}(\epsilon)t_l^s(\epsilon)\mathbf{B}_{li}(\epsilon)t_i^s(\epsilon) - \mathbf{B}_{il}(\epsilon)t_l^s(\epsilon)A_{lk}(\epsilon)t_k^s(\epsilon)A_{kj}(\epsilon)t_j^s(\epsilon)A_{ji}(\epsilon)t_i^s(\epsilon)$$
$$+A_{ij}(\epsilon)t_j^s(\epsilon)A_{jk}(\epsilon)t_k^s(\epsilon)\mathbf{B}_{kl}(\epsilon)t_l^s(\epsilon)A_{li}(\epsilon)t_i^s(\epsilon) - A_{il}(\epsilon)t_l^s(\epsilon)\mathbf{B}_{lk}(\epsilon)t_k^s(\epsilon)A_{kj}(\epsilon)t_j^s(\epsilon)A_{ji}(\epsilon)t_i^s(\epsilon)\}, \quad (11)$$

and

$$\boldsymbol{\mathcal{K}}_{i\bar{j}kl} = \frac{1}{2\pi}\mathrm{Re}\ \mathrm{Tr}\int_{-\infty}^{\epsilon_F}\mathrm{d}\epsilon\,\{\mathbf{B}_{ij}(\epsilon)t_j^s(\epsilon)A_{jk}(\epsilon)t_k^s(\epsilon)A_{kl}(\epsilon)t_l^s(\epsilon)A_{li}(\epsilon)t_i^s(\epsilon) - A_{il}(\epsilon)t_l^s(\epsilon)A_{lk}(\epsilon)t_k^s(\epsilon)A_{kj}(\epsilon)t_j^s(\epsilon)\mathbf{B}_{ji}(\epsilon)t_i^s(\epsilon)$$
$$+A_{ij}(\epsilon)t_j^s(\epsilon)\mathbf{B}_{jk}(\epsilon)t_k^s(\epsilon)A_{kl}(\epsilon)t_l^s(\epsilon)A_{li}(\epsilon)t_i^s(\epsilon) - A_{il}(\epsilon)t_l^s(\epsilon)A_{lk}(\epsilon)t_k^s(\epsilon)\mathbf{B}_{kj}(\epsilon)t_j^s(\epsilon)A_{ji}(\epsilon)t_i^s(\epsilon)\}. \quad (12)$$

In general, the vector parameters $\boldsymbol{\mathcal{K}}_{ijk\bar{l}}$ and $\boldsymbol{\mathcal{K}}_{i\bar{j}kl}$ are not identical. The indices $\bar{j}$ and $\bar{l}$, typeset with a bar, are the sites at which on-site dot products with the unit vectors $\mathbf{S}$ are formed. The total spin-chiral contribution obtained from the fourth-order term in the Taylor expansion has the following form:

$$E^{\mathrm{SC}} = -\sum_{ijkl}\left\{\left(\boldsymbol{\mathcal{K}}_{ijk\bar{l}}\cdot\mathbf{S}_l\right)\left[\mathbf{S}_i\cdot\left(\mathbf{S}_j\times\mathbf{S}_k\right)\right] + \left(\boldsymbol{\mathcal{K}}_{i\bar{j}kl}\cdot\mathbf{S}_j\right)\left[\mathbf{S}_i\cdot\left(\mathbf{S}_k\times\mathbf{S}_l\right)\right]\right\}. \quad (13)$$

Note that the direction of $\boldsymbol{\mathcal{K}}$ is determined by a linear combination of $\boldsymbol{\mathcal{B}}$ vectors. For the crystal structure of interest, where the Mn atoms form equilateral triangular plaquettes, the components of $\boldsymbol{\mathcal{K}}$ within each plaquette vanish by symmetry. Furthermore, E^{SC} can be written as

$$E^{\mathrm{SC}} = -\sum_{ijkl}(\boldsymbol{\mathcal{K}}_{ijk\bar{l}}\cdot\mathbf{S}_l)\chi_{ijk} + (\boldsymbol{\mathcal{K}}_{i\bar{j}kl}\cdot\mathbf{S}_j)\chi_{ikl} \quad (14)$$
$$= -\sum_{ijkl}(\boldsymbol{\mathcal{K}}_{ijk\bar{l}}\cdot\mathbf{S}_l)L^{\mathrm{TO}}_{ijk}\gamma^{\mathrm{TO}}_{ijk} + (\boldsymbol{\mathcal{K}}_{i\bar{j}kl}\cdot\mathbf{S}_j)L^{\mathrm{TO}}_{ikl}\gamma^{\mathrm{TO}}_{ikl} \quad (15)$$
$$= -\sum_{ijkl}\gamma^{\mathrm{TO}}_{ijk}\mathcal{K}_{ijk\bar{l}}(\mathbf{L}_{ijk\bar{l}}\cdot\mathbf{S}_l) + \gamma^{\mathrm{TO}}_{ikl}\mathcal{K}_{i\bar{j}kl}(\mathbf{L}_{i\bar{j}kl}\cdot\mathbf{S}_j), \quad (16)$$

where $\mathbf{L}_{ijk\bar{l}} = \hat{\boldsymbol{\mathcal{K}}}_{ijk\bar{l}}L^{\mathrm{TO}}_{ijk}$, $\mathbf{L}_{i\bar{j}kl} = \hat{\boldsymbol{\mathcal{K}}}_{i\bar{j}kl}L^{\mathrm{TO}}_{ikl}$ and γ^{TO} is the inverse susceptibility connecting the 3-spin scalar chirality to the topological orbital moment. Finally we can recover the form given in the main manuscript:

$$E^{\mathrm{SC}} = -\sum_l \varkappa_l(\boldsymbol{\mathcal{L}}_l\cdot\mathbf{S}_l) - \sum_j \bar{\varkappa}_j(\boldsymbol{\mathcal{L}}_j\cdot\mathbf{S}_j) = -\sum_m \varkappa^{\mathrm{SC}}_m(\boldsymbol{\mathcal{L}}_m\cdot\mathbf{S}_m), \quad (17)$$

with $\varkappa_l\boldsymbol{\mathcal{L}}_l = \sum_{ijk}\gamma^{\mathrm{TO}}_{ijk}\mathcal{K}_{ijk\bar{l}}\mathbf{L}_{ijk\bar{l}}$, $\bar{\varkappa}_j\boldsymbol{\mathcal{L}}_j = \sum_{ikl}\gamma^{\mathrm{TO}}_{ikl}\mathcal{K}_{i\bar{j}kl}\mathbf{L}_{i\bar{j}kl}$ and $\varkappa^{\mathrm{SC}} = \varkappa - \bar{\varkappa}$. Our first-principles simulations indicate that for MnGe and FeGe, the direction of $\boldsymbol{\mathcal{L}}_m$ coincides with that of $\boldsymbol{L}^{\mathrm{TO}}_m$ and therefore:

$$E^{\mathrm{SC}} = -\sum_i \varkappa^{\mathrm{SC}}_i(\boldsymbol{L}^{\mathrm{TO}}_i\cdot\mathbf{S}_i). \quad (18)$$

The sixth order term obtained from Supplementary Eq. (1) involves a plaquette of six magnetic moments. We expect the largest contribution to be the isotropic, rotationally invariant one, as this term can be finite without spin-orbit interaction, which gives rise to the chiral-chiral interaction

$$\delta E^{\mathrm{CC}}_{ijklmn} = -\varkappa^{\mathrm{CC}}_{ijklmn}\left[\delta\mathbf{S}_i\cdot\left(\delta\mathbf{S}_j\times\delta\mathbf{S}_k\right)\right]\left[\delta\mathbf{S}_l\cdot\left(\delta\mathbf{S}_m\times\delta\mathbf{S}_n\right)\right] \quad (19)$$

of the topological-chiral interaction. We expect the largest contribution to E^{CC} to come from a plaquette made of three neighboring atoms. In that case one finds:

$$\delta E^{\mathrm{CC}}_{ijkijk} = -\varkappa^{\mathrm{CC}}_{ijkijk}\left[\delta\mathbf{S}_i\cdot\left(\delta\mathbf{S}_j\times\delta\mathbf{S}_k\right)\right]\left[\delta\mathbf{S}_i\cdot\left(\delta\mathbf{S}_j\times\delta\mathbf{S}_k\right)\right]. \quad (20)$$

In the main text we simplified the notation of $\varkappa^{\mathrm{CC}}_{ijkijk}$ to $\varkappa^{\mathrm{CC}}_{ijk}$. Although this six-order term of the total energy with respect the rotation of three magnetic moments sounds like a tiny correction term, and this is indeed rather unexpected, we have shown in the main text that for MnGe in difference to FeGe, this contribution can be surprisingly large, larger than the Dzyaloshinskii-Moriya interaction. In general, the effective 6-spin interaction is given by

$$\varkappa^{\mathrm{CC}}_{ijklmn} = \frac{1}{3\pi}\mathrm{Im}\ \mathrm{Tr}\int_{-\infty}^{\epsilon_F}\mathrm{d}\epsilon\,A_{ij}(\epsilon)t_j^s(\epsilon)A_{jk}(\epsilon)t_k^s(\epsilon)A_{kl}(\epsilon)t_l^s(\epsilon)A_{lm}(\epsilon)t_m^s(\epsilon)A_{mn}(\epsilon)t_n^s(\epsilon)A_{ni}(\epsilon)t_i^s(\epsilon). \quad (21)$$

We have derived the SCI as a fourth order term of the total energy expansion with respect to concerted infinitesimal rotations of the spin-magnetic moment linear in spin-orbit interaction and the CCI as the isotropic sixth-order contribution. In many cases these terms might be small. It is worth pointing out that the second, fourth and six order terms are proportional to powers t^2, t^4, t^6, respectively, of the scattering matrix and thus proportional to powers S^2, S^4, S^6 of the size of the spin-moment. Thus, the topological-chiral interaction might be more prevalent in magnetic systems with large magnetic moments. This is consistent with the analysis of Hoffmann *et al.* [7] for quantum spin-systems, where the terms in Supplementary Eqs. (13) and (20) require a spin-1 magnet. To separate the role of the electronic structure due to the interference of Bloch wavefunctions over many sites of the underlying lattice from the properties of local atomic moments on atom sites, it might be useful to scale the interaction parameters $\varkappa^{\mathrm{CC}}$ as $\bar{\varkappa}^{\mathrm{CC}}S^6$ and $\boldsymbol{\mathcal{K}}$ as $\bar{\boldsymbol{\mathcal{K}}}S^4$. Then, the interaction parameters $\bar{\varkappa}^{\mathrm{CC}}$ and $\bar{\boldsymbol{\mathcal{K}}}$ might be more comparable across different systems.

Supplementary Note 2 | Generalization of chiral energy expression

The chiral-chiral interaction, the topological orbital moment, and the spin-chiral interaction, see (2)–(4), respectively, in the main text, have been formulated for the considered cubic B20 crystals as one-parameter theories. For more general cases, starting from the paradigm that the scalar spin chirality gives rise to a topological orbital moment, we motivate here an alternative to our rigorous derivation in Supplementary Note 1 in terms of intuitive expressions for the topological-chiral magnetic interactions, where the effective interaction constants take the form of tensor quantities. Specifically, we can express the emergent magnetic field $\mathbf{B}_i^{\mathrm{eff}}$ that roots in the finite scalar spin chirality $\chi_{ijk} = \mathbf{S}_i \cdot (\mathbf{S}_j \times \mathbf{S}_k)$ as $\mathbf{B}_i^{\mathrm{eff}} = \mu_i^{\mathrm{TO}} \sum_{(jk)} \boldsymbol{\chi}_{ijk}$, where the sum is restricted to triangles including the ith site, μ_i^{TO} is a real scalar, and $\boldsymbol{\chi}_{ijk} = \chi_{ijk}\boldsymbol{\tau}_{ijk}$. The unit vector $\boldsymbol{\tau}_{ijk} \propto (\mathbf{R}_j - \mathbf{R}_i) \times (\mathbf{R}_k - \mathbf{R}_i)$ denotes the normal of the triangle formed by the spins at positions $\mathbf{R}_i$, $\mathbf{R}_j$, and $\mathbf{R}_k$. As a consequence, the energy due to the proposed chiral-chiral interaction assumes the universal system-independent structure

$$
\begin{aligned}
E^{\mathrm{CC}} &= -\frac{1}{2}\sum_{ii'} \left(\mathbf{B}_i^{\mathrm{eff}}\right)^{\dagger} \underline{\underline{\varkappa}}_{ii'}^{\mathrm{TO}} \mathbf{B}_{i'}^{\mathrm{eff}} = -\frac{1}{2}\sum_{ii'}\sum_{(jk)}\sum_{(j'k')} \boldsymbol{\chi}_{ijk}^{\dagger} \left(\mu_i^{\mathrm{TO}} \underline{\underline{\varkappa}}_{ii'}^{\mathrm{TO}} \mu_{i'}^{\mathrm{TO}}\right) \boldsymbol{\chi}_{i'j'k'} \\
&= -\frac{1}{2}\sum_{ii'}\sum_{(jk)}\sum_{(j'k')} \boldsymbol{\chi}_{ijk}^{\dagger} \underline{\underline{\varkappa}}_{ii'}^{\mathrm{CC}} \boldsymbol{\chi}_{i'j'k'} = -\frac{1}{2}\sum_{ii'}\sum_{(jk)}\sum_{(j'k')} \left[\mathbf{S}_i \cdot (\mathbf{S}_j \times \mathbf{S}_k)\right] \boldsymbol{\tau}_{ijk}^{\dagger} \underline{\underline{\varkappa}}_{ii'}^{\mathrm{CC}} \boldsymbol{\tau}_{i'j'k'} \left[\mathbf{S}_{i'} \cdot (\mathbf{S}_{j'} \times \mathbf{S}_{k'})\right] .
\end{aligned} \tag{22}
$$

Here, the tensor $\underline{\underline{\varkappa}}_{ii'}^{\mathrm{CC}} = \mu_i^{\mathrm{TO}} \underline{\underline{\varkappa}}_{ii'}^{\mathrm{TO}} \mu_{i'}^{\mathrm{TO}}$ encodes the magnitude and symmetry of the chiral-chiral coupling. In fact, the general Supplementary Eq. (22) is intimately connected to the topological orbital moment as the first derivative of E^{CC} with respect to the emergent magnetic field. The topological orbital moment is the linear response $\mathbf{L}_i^{\mathrm{TO}} = \underline{\underline{\chi}}^{\mathrm{TO}} \mathbf{B}_i^{\mathrm{eff}}$ to the emergent magnetic field as mediated by the tensor $\underline{\underline{\chi}}^{\mathrm{TO}}$ of the topological orbital susceptibility. Thus, it follows the expression

$$
\mathbf{L}_i^{\mathrm{TO}} = \mu_i^{\mathrm{TO}} \underline{\underline{\chi}}^{\mathrm{TO}} \sum_{(jk)} \boldsymbol{\chi}_{ijk} = \mu_i^{\mathrm{TO}} \underline{\underline{\chi}}^{\mathrm{TO}} \sum_{(jk)} \left[\mathbf{S}_i \cdot (\mathbf{S}_j \times \mathbf{S}_k)\right] \boldsymbol{\tau}_{ijk} . \tag{23}
$$

Finally, the energy contribution due to the postulated spin-chiral interaction, which is given by Eq. (4) of the main text for the B20 structure can be as well represented for more general case using tensorial notation

$$
\begin{aligned}
E^{\mathrm{SC}} = -\sum_{ii'} \varkappa_{ii'}^{\mathrm{SC}} \left(\mathbf{L}_i^{\mathrm{TO}}\right)^{\dagger} \cdot \mathbf{S}_{i'} &= -\sum_{ii'}\sum_{(jk)} \boldsymbol{\chi}_{ijk}^{\dagger} \left(\varkappa_{ii'}^{\mathrm{SC}} \mu_i^{\mathrm{TO}} \underline{\underline{\chi}}^{\mathrm{TO},\dagger}\right) \mathbf{S}_{i'} \\
&= -\sum_{ii'}\sum_{(jk)} \boldsymbol{\chi}_{ijk}^{\dagger} \underline{\underline{\varkappa}}_{ii'}^{\mathrm{SC}} \mathbf{S}_{i'} = -\sum_{ii'}\sum_{(jk)} \left[\mathbf{S}_i \cdot (\mathbf{S}_j \times \mathbf{S}_k)\right] \boldsymbol{\tau}_{ijk}^{\dagger} \underline{\underline{\varkappa}}_{ii'}^{\mathrm{SC}} \mathbf{S}_{i'}
\end{aligned} \tag{24}
$$

with the coupling matrix $\underline{\underline{\varkappa}}_{ii'}^{\mathrm{SC}} = \varkappa_{ii'}^{\mathrm{SC}} \mu_i^{\mathrm{TO}} \underline{\underline{\chi}}^{\mathrm{TO},\dagger}$, which directly relates to the tensor of the topological orbital susceptibility.

Supplementary Note 3 | Effective spin-lattice Hamiltonian

With the discovered topological-chiral interactions we propose the following extended spin-lattice Hamiltonian consisting of Heisenberg, symmetric fourth-order [7], Dzyaloshinskii–Moriya [6], and Zeeman interaction together with chiral-chiral interaction, the magnetic anisotropy and the spin-chiral interaction to describe the static, dynamical and thermodynamical magnetic

properties of advanced magnetic materials

$$
\begin{aligned}
H = & -\sum_{ij} J_{ij}\mathbf{S}_i \cdot \mathbf{S}_j - \sum_{ijkl} K_{ijkl}(\mathbf{S}_i \cdot \mathbf{S}_j)(\mathbf{S}_k \cdot \mathbf{S}_l) - \sum_{ij} \mathbf{D}_{ij} \cdot (\mathbf{S}_i \times \mathbf{S}_j) - \sum_i \mathbf{B} \cdot \mathbf{S}_i \\
& - \frac{1}{2} \sum_{ii'} \sum_{(jk)} \sum_{(j'k')} \left[\mathbf{S}_i \cdot (\mathbf{S}_j \times \mathbf{S}_k)\right] \boldsymbol{\tau}^{\dagger}_{ijk} \underline{\underline{\varkappa}}^{\mathrm{CC}}_{ii'} \boldsymbol{\tau}_{i'j'k'} \left[\mathbf{S}_{i'} \cdot (\mathbf{S}_{j'} \times \mathbf{S}_{k'})\right] \\
& - \sum_{ij} \mathbf{S}_i \underline{\underline{A}}_{ij} \mathbf{S}_j - \sum_{ii'} \sum_{(jk)} \left[\mathbf{S}_i \cdot (\mathbf{S}_j \times \mathbf{S}_k)\right] \boldsymbol{\tau}^{\dagger}_{ijk} \underline{\underline{\varkappa}}^{\mathrm{SC}}_{ii'} \mathbf{S}_{i'} \,,
\end{aligned}
\tag{25}
$$

with microscopic interactions parameters, J_{ij}, K_{ijkl}, $\mathbf{D}_{ij}$, $\underline{\underline{\varkappa}}^{\mathrm{CC}}_{ii'}$, $\underline{\underline{A}}_{ij}$, $\underline{\underline{\varkappa}}^{\mathrm{SC}}_{ii'}$ that can be determined by first-principles theory as exemplified in this work, and describe the strength of the corresponding interaction. $\mathbf{B}$ denotes the external magnetic field. i, j, k, l denotes lattice sites. Besides the well-known pair interactions J_{ij}, $\mathbf{D}_{ij}$, $\underline{\underline{A}}_{ij}$, for symmetric and antisymmetric exchange, and the magnetocrystalline anisotropy, which scale proportionally to zeroth, first and at least second order in spin-orbit interaction, we propose interaction terms that originate from the scalar spin chirality, namely, the chiral-chiral coupling tensor as encoded in $\underline{\underline{\varkappa}}^{\mathrm{CC}}_{ii'}$, and the spin-chiral interaction quantified by tensor quantity $\underline{\underline{\varkappa}}^{\mathrm{SC}}_{ii'}$. Regarding the chiral-chiral and spin-chiral interactions mediated by tensors $\underline{\underline{\varkappa}}^{\mathrm{CC}}_{ii'}$ and $\underline{\underline{\varkappa}}^{\mathrm{SC}}_{ii'}$, respectively, we generally anticipate that the non-local terms, $i \neq i'$ are small as compared to the dominant local contribution with $i = i'$. The discussion bears similarities to the discussion of the single-ion, $\underline{\underline{A}}_{ii}$, versus two-site magnetic anisotropy $\underline{\underline{A}}_{ij}$ with $i \neq j$. The latter is typically neglected.

Evaluating Supplementary Eq. (25) for the cubic B20 compounds with antiferromagnetic spin configuration, we arrive in the main text at analytic expressions for the angular dependence of the total energy. In particular, the total energy contains a constant contribution $E_0^{\mathrm{ex}} = J$ due to Heisenberg coupling, the term $E_0^{\mathrm{4th}} = K(\sin^2 2\theta + \sin^4\theta \sin^2 2\phi) + K'$ for the 4th-order exchange, and the contribution $E_0^{\mathrm{DM}} = D(\sin^2\theta \sin 2\phi + \sin 2\theta\,(\cos\phi + \sin\phi))$ due to DMI. Note that the 4th-order exchange combines the common biquadratic interaction with 4-spin–3-site and 4-spin–4-site interactions [7]. Moreover, in non-collinear B20 magnets, the single-site anisotropy contains non-trivial uniaxial and cubic contributions, both of which are negligible as compared to the proposed magnetic interactions, see Supplementary Note 5 and Supplementary Table 1. In case of the considered B20 materials, the four magnetic atoms are symmetry-related, rendering additionally the chiral-chiral and spin-chiral response independent of the site index i. Finally, since the point group of the B20 structure is cubic, we demonstrate in the main text that the energetics of the spin system due to the novel topological-chiral interactions are described excellently by single scalars $\varkappa^{\mathrm{CC}}$ and $\varkappa^{\mathrm{SC}}$. We uncover the importance of higher-order exchange interactions in the B20 germanides by fitting the analytic expressions to the first-principles results for the total energy. Supplementary Table 1 summarizes the exchange parameters that we obtained and used in the main text, assuming a constant size of the spin magnetic moment.

Supplementary Note 4 | Variations of spin magnetic moment

As shown in the top panels of Supplementary Figure 1, the size of the spin magnetic moments changes by up to 12% in FeGe and 3% in MnGe as a function of the magnetic configuration. In the absence of spin-orbit coupling, we examine the impact of these variations on the considered Heisenberg coupling, 4th-order exchange, and the proposed chiral-chiral interaction. Supplementary Figure 1 illustrates the modulation of the magnetic moment with θ for fixed ϕ, which can be described by a function $g_\phi(\theta)$ via

$$
|\mathbf{S}_i(\theta,\phi)| = g_\phi(\theta) S \,, \tag{26}
$$

where S is the size of the local spin moment at $\theta = 0°$. We express the modulating function $g_\phi(\theta)$ as

$$
g_\phi(\theta) = 1 - \sum_{n=1}^{N} c_\phi^n \sin^2(n\theta) \,, \tag{27}
$$

where c_ϕ^n is a fitting parameter, and the finite cutoff N restricts the summation. As a consequence, the changes in the length of the spin magnetic moment modify the angular dependence of the total-energy contributions according to

$$
\Delta E^{\mathrm{ex}}(\theta,\phi) = [g_\phi^2(\theta) - 1]\, E_0^{\mathrm{ex}}(\theta,\phi) \,, \tag{28}
$$

$$
\Delta E^{\mathrm{4th}}(\theta,\phi) = [g_\phi^4(\theta) - 1]\, E_0^{\mathrm{4th}}(\theta,\phi) \,, \tag{29}
$$

$$
\Delta E^{\mathrm{CC}}(\theta,\phi) = [g_\phi^6(\theta) - 1]\, E_0^{\mathrm{CC}}(\theta,\phi) \,, \tag{30}
$$

where E_0^{ex}, E_0^{4th}, and E_0^{CC} are, respectively, the Heisenberg exchange, 4th-order exchange, and chiral-chiral contributions to the energy obtained under the assumption of a constant length of the spin magnetic moment (see main text and Supplementary Note 6). Using the coefficients shown in Supplementary Table 2, we find that taking into account the variation of the spin-moment length improves the fit to the first-principles results for the dependence of the total energy on the magnetic configuration, see Supplementary Figure 1. This underlines the importance of the chiral-chiral interaction to describe MnGe.

Supplementary Note 5 | Single-site magnetocrystalline anisotropy

In this Supplementary Note, we assess the overall importance of the single-site magnetocrystalline anisotropy (MCA) in the B20 chiral magnets. In terms of simple symmetry considerations, we demonstrate that the studied systems exhibit to lowest order a non-trivial uniaxial anisotropy, owing to the locally broken cubic symmetry in combination with non-collinear magnetism. These finding we fully confirm by first-principles calculations.

Symmetry analysis. The energy of MCA can be expressed in terms of the local magnetic moment $\mathbf{S}_i$ at site i [10]

$$E^{\mathrm{MCA}} = -\sum_{i,\alpha\beta} \underline{\underline{A}}^{(2)}_{i,\alpha\beta} S_{i,\alpha} S_{i,\beta} - \sum_{i,\alpha\beta\gamma\delta} \underline{\underline{A}}^{(4)}_{i,\alpha\beta\gamma\delta} S_{i,\alpha} S_{i,\beta} S_{i,\gamma} S_{i,\delta} - \dots , \tag{31}$$

and where Greek subscripts stand for the Cartesian coordinates x, y, and z. The tensors $\underline{\underline{A}}_i^{(r)}$ of rank r describe the energy contributions at different orders, and the non-zero tensor elements are determined by the local symmetries at site i as given by the site-symmetry group (i.e., set of all symmetry operations of the space group of the crystal that leave site i invariant). While the series expansion in Supplementary Eq. (31) can be extended to higher orders, we neglect those terms in the following as the contributions are anticipated to be negligibly small. According to Neumann's principle, the local quantities $\underline{\underline{A}}_i^{(r)}$ need to obey the local symmetries at a given site. This is used to reduce the number of independent tensor elements via the following relations:

$$\underline{\underline{A}}^{(2)}_{i,\alpha\beta} = \sum_{\alpha'\beta'} \underline{\underline{M}}_{i,\alpha\alpha'} \underline{\underline{M}}_{i,\beta\beta'} \underline{\underline{A}}^{(2)}_{i,\alpha'\beta'} \tag{32}$$

$$\underline{\underline{A}}^{(4)}_{i,\alpha\beta\gamma\delta} = \sum_{\alpha'\beta'\gamma'\delta'} \underline{\underline{M}}_{i,\alpha\alpha'} \underline{\underline{M}}_{i,\beta\beta'} \underline{\underline{M}}_{i,\gamma\gamma'} \underline{\underline{M}}_{i,\delta\delta'} \underline{\underline{A}}^{(4)}_{i,\alpha'\beta'\gamma'\delta'} , \tag{33}$$

which need to be fulfilled for all symmetry elements of the site-symmetry group $\mathcal{G}$. The transformation matrix $\underline{\underline{M}}_i(\mathcal{G})$ corresponds to the matrix representation of the symmetry operations that leave site i invariant. In the case of simple magnetic materials crystallizing in the fcc or bcc lattice, these local symmetries are very high, which renders the 2nd-order anisotropy tensor proportional to the identity matrix [11], giving rise to a constant energy contribution in Supplementary Eq. (31) irrespective of the underlying magnetic structure.

In the considered B20 systems, however, the local symmetries are reduced such that each site i has only one local symmetry direction $\mathbf{n}_i$, which is a three-fold rotational axis that points along one of the cube diagonals $(1,1,1)$, $(-1,-1,1)$, $(-1,1,-1)$, and $(1,-1,-1)$, see Figure 2a of the main text. Although the on-site anisotropy tensors $\underline{\underline{A}}_i^{(r)}$ are thus not identical for different i, they are related as the magnetic sublattices are connected by non-symmorphic symmetries of the space group $P2_13$. Following Neumann's principle as stated in Supplementary Eq. (32), we then arrive at the 2nd-order anisotropy tensor

$$\underline{\underline{A}}_i^{(2)} = \begin{pmatrix} a & b\, n_{i,z} & c\, n_{i,y} \\ c\, n_{i,z} & a & b\, n_{i,x} \\ b\, n_{i,y} & c\, n_{i,x} & a \end{pmatrix} , \tag{34}$$

where the site-independent coefficients a, b, and c are the only free parameters. Using this form in Supplementary Eq. (31) and summing over the four magnetic atoms in the B20 unit cell, we obtain the 2nd-order contribution to the anisotropy energy:

$$E^{\mathrm{MCA},(2)} = -\sum_{i=1}^{4} \sum_{\alpha\beta} \underline{\underline{A}}^{(2)}_{i,\alpha\beta} S_{i,\alpha} S_{i,\beta} = -A^{(2)} \sum_{i=1}^{4} \left(\mathbf{n}_i \cdot \mathbf{S}_i\right)^2 , \tag{35}$$

where $A^{(2)} = (b+c)/2$ is an effective anisotropy parameter. While this expression amounts to a constant in collinear cubic magnets, it describes a non-trivial uniaxial anisotropy mediated by the local symmetry axes $\mathbf{n}_i$ in B20 compounds with non-collinear magnetic texture. The local symmetry direction $\mathbf{n}_i$ pointing along the main-diagonal of the cubic unit cell define local easy ($A^{(2)} > 0$) or hard ($A^{(2)} < 0$) axes depending on the sign of $A^{(2)}$.

Following the same rationale by using the local three-fold rotations in Supplementary Eq. (33), we arrive at the 4th-order energy contribution to the magnetocrystalline anisotropy:

$$\begin{aligned} E^{\mathrm{MCA},(4)} &= -\sum_{i=1}^{4} \sum_{\alpha\beta\gamma\delta} \underline{\underline{A}}^{(4)}_{i,\alpha\beta\gamma\delta} S_{i,\alpha} S_{i,\beta} S_{i,\gamma} S_{i,\delta} \\ &= -\sum_{i=1}^{4} \sum_{\alpha\neq\beta\neq\gamma} \left[A_1^{(4)} S_{i,\alpha}^2 S_{i,\beta}^2 + A_2^{(4)} (\mathbf{n}_{i,\alpha}^2 \mathbf{S}_{i,\alpha}^2)(\mathbf{n}_{i,\beta} \cdot \mathbf{S}_{i,\beta})(\mathbf{n}_{i,\gamma} \cdot \mathbf{S}_{i,\gamma}) + \left(A_3^{(4)} + A_4^{(4)} \varepsilon_{\alpha\beta\gamma}\right)(\mathbf{n}_{i,\alpha}^3 \cdot \mathbf{S}_{i,\alpha}^3)(\mathbf{n}_{i,\beta} \cdot \mathbf{S}_{i,\beta}) \right], \end{aligned} \tag{36}$$

where $A_i^{(4)}$ are effective anisotropy constants and $\varepsilon_{\alpha\beta\gamma}$ is the antisymmetric 3D Levi-Civita symbol. Since the first term in Supplementary Eq. (36) is independent of $\mathbf{n}_i$, it can be identified with the well-known cubic anisotropy, whereas the other contributions are non-cubic corrections due to the locally reduced symmetry. The non-cubic terms become active only in non-collinear systems but vanish in B20 ferromagnets, which are correspondingly characterized by the 4th-order cubic anisotropy only.

First-principles results. To disentangle the MCA contribution from other spin-orbit effects in our first-principles calculations, we consider the magnetic configuration described by Eq. 4 of the main text with $\theta = 90°$ and $\phi = 45°$, and rotate the resulting coplanar texture by an angle α around the z axis (see also Supplementary Figure 2a). Such a rotation of the magnetic structure by an angle α implies that the angle β_i formed between each magnetic moment $\mathbf{S}_i$ and the local three-fold rotation axis $\mathbf{n}_i$ changes by $\beta_i = \arccos[\sqrt{2/3}\cos\alpha]$. As the Dzyaloshinskii-Moriya and spin-chiral interactions are independent of the rotation angle, any variation of the total energy, $\Delta E^{\mathrm{SOI}}(\alpha)$, with α originates from the MCA. By using Supplementary Eqs. (35) and (36) for the considered magnetic structure, we find that the change of the spin-orbit energy assumes the form

$$\Delta E^{\mathrm{SOI}}(\alpha) = E^{\mathrm{an}}(\alpha) - E^{\mathrm{an}}(0) = A_{\mathrm{uniax}} \sin^2\alpha + A_{\mathrm{cubic}} \sin^2 2\alpha\,, \tag{37}$$

where $A_{\mathrm{uniax}} = -2(4A^{(2)} + A_3^{(4)} + A_4^{(4)})$ and $A_{\mathrm{cubic}} = -A_1^{(4)}$ directly relate to the non-cubic and cubic terms of the anisotropy as given by Supplementary Eqs. (35) and (36), respectively. We obtain these material-specific anisotropy constants by fitting the above model to our first-principles data shown in Supplementary Figure 2b for the examples of FeGe and MnGe. As a result, we find that the non-cubic corrections A_{uniax}, which amount to −0.03 meV/uc for FeGe and −0.78 meV/uc for MnGe, dominate by far over the tiny cubic anisotropy of $|A_{\mathrm{cubic}}| < 0.002$ meV/uc, while the abbreviation uc stands for unit cell. While the non-cubic corrections to the magneto-crystalline anisotropy has been largely ignored based on arguments of the cubic crystal structure of B20 materials that hold, however, only true in case of collinear magnetism, in comparison to the magnitude of the other spin-orbit effects discussed in the main text, also the non-cubic anisotropy in non-collinear B20 magnets is negligibly small, see Supplementary Table 1.

It is worth noting that in noncollinear B20 magnets, the MAE and SCI defines the orientation of the complex of four spins (rather than one local spin) with respect to the crystallographic axes. In a noncollinear system, this complex with fixed mutual orientation of spins is equivalent to a solid body (described with at least three vectors, see Supplementary Note 7), while the structure of a collinear magnet can be reduced to the orientation of one vector. Thus, the MAE expansion for noncollinear magnets starts from the second order terms in spin variables even in high-symmetry (cubic) crystals which is associated with a larger value of anisotropy in such systems.

Supplementary Note 6 | Topological-chiral magnetic interactions for smooth magnetization fields

In this Supplementary Note we present a continuum representation ($\mathbf{S}_i \to \mathbf{m}(\mathbf{r})$) of the topological-chiral magnetic interactions, i.e. topological orbital moment, the chiral-chiral and the spin-chiral interaction. We start with the scalar spin-chirality defined in the spin-lattice model as $\chi_{ijk} = \mathbf{S}_i \cdot (\mathbf{S}_j \times \mathbf{S}_k)$, which is the scalar triple product of spins at vertices of a triangular plaquette of atoms defined by the positions of atoms $\mathbf{R}_{i,j,k}$ at sites i, j, and k. In continuum theory, we change from site-dependent quantities to densities in the volume element d$\mathbf{r}$. We associate this volume element with site i and express the sites j and k of our triangular plaquettes relative to site i.

The scalar spin-chirality can be rewritten in the form

$$\chi_{ijk} = \mathbf{S}_i \cdot (\mathbf{S}_j - \mathbf{S}_i) \times (\mathbf{S}_k - \mathbf{S}_i) = \chi_{i,j-i,k-i} = \chi_{i,ji,ki}\,. \tag{38}$$

The latter definition was chosen for convenience. Expressing the atom position at site j and k relative to i, $\mathbf{R}_j = \mathbf{R}_i + R_{ji}\hat{\mathbf{e}}_{ji}$ and $\mathbf{R}_k = \mathbf{R}_i + R_{ki}\hat{\mathbf{e}}_{ki}$, where $\hat{\mathbf{e}}$ is the unit direction to the neighboring site of the triangle, *e.g.* $\hat{\mathbf{e}}_{ji} = (\mathbf{R}_j - \mathbf{R}_i)/|\mathbf{R}_j - \mathbf{R}_i|$, we can

expand the magnetization density $\mathbf{m}(\mathbf{r})$ as

$$\mathbf{m}(\mathbf{R}_j) = \mathbf{m}(\mathbf{R}_i + R_{ji}\,\hat{\mathbf{e}}_{ji}) \simeq \mathbf{m}(\mathbf{R}_i) + R_{ji}\,\nabla\mathbf{m}\,\hat{\mathbf{e}}_{ji} = \mathbf{m}(\mathbf{R}_i) + R_{ji}\sum_{\alpha}\frac{\partial\mathbf{m}}{\partial r_\alpha}\,\hat{\mathbf{e}}_{\alpha|ji} \tag{39}$$

and accordingly

$$\mathbf{m}(\mathbf{R}_k) = \mathbf{m}(\mathbf{R}_i + R_{ki}\,\hat{\mathbf{e}}_{ki}) \simeq \mathbf{m}(\mathbf{R}_i) + R_{ki}\,\nabla\mathbf{m}\,\hat{\mathbf{e}}_{ki} = \mathbf{m}(\mathbf{R}_i) + R_{ki}\sum_{\beta}\frac{\partial\mathbf{m}}{\partial r_\beta}\,\hat{\mathbf{e}}_{\beta|ki}\,, \tag{40}$$

where R_{ji} and R_{ki} are distances between sites i and j(k) in units of lattice parameters and $\alpha, \beta \in \{x, y, z\}$. Inserting this into Supplementary Eq. (38) we obtain

$$\chi_{ijk} \simeq R_{ji}R_{ki}\sum_{\alpha,\beta}\mathbf{m}\cdot\left(\frac{\partial\mathbf{m}}{\partial r_\alpha}\times\frac{\partial\mathbf{m}}{\partial r_\beta}\right)\bigg|_{\mathbf{r}=\mathbf{R}_i}\hat{\mathbf{e}}_{\alpha|ji}\,\hat{\mathbf{e}}_{\beta|ki}\,. \tag{41}$$

For convenience we define

$$f_{\alpha\beta} = \mathbf{m}\cdot\left(\frac{\partial\mathbf{m}}{\partial r_\alpha}\times\frac{\partial\mathbf{m}}{\partial r_\beta}\right) \quad \text{with} \quad f_{\alpha\beta} = -f_{\beta\alpha} \quad \text{and} \quad f_{\alpha\beta} = 0 \text{ for } \alpha = \beta\,. \tag{42}$$

With this definition we can simplify Supplementary Eq. (41)

$$\begin{aligned}\chi_{ijk} &\simeq R_{ji}R_{ki}\sum_{\alpha,\beta>\alpha} f_{\alpha\beta}\,(\hat{\mathbf{e}}_{\alpha|ji}\,\hat{\mathbf{e}}_{\beta|ki} - \hat{\mathbf{e}}_{\beta|ji}\,\hat{\mathbf{e}}_{\alpha|ki})\\ &= \frac{1}{2}R_{ji}R_{ki}\sum_{\alpha,\beta} f_{\alpha\beta}\,(\hat{\mathbf{e}}_{\alpha|ji}\,\hat{\mathbf{e}}_{\beta|ki} - \hat{\mathbf{e}}_{\beta|ji}\,\hat{\mathbf{e}}_{\alpha|ki})\\ &= \frac{1}{2}R_{ji}R_{ki}\sum_{\alpha,\beta}\sum_{\mu,\nu} f_{\alpha\beta}\,(\delta_{\alpha\mu}\delta_{\beta\nu} - \delta_{\alpha\nu}\delta_{\beta\mu})\hat{\mathbf{e}}_{\mu|ji}\,\hat{\mathbf{e}}_{\nu|ki}\end{aligned} \tag{43}$$

making use of the contracted epsilon identity, $\sum_{\gamma=1}^{3}\varepsilon_{\gamma\alpha\beta}\varepsilon_{\gamma\mu\nu} = \delta_{\alpha\mu}\delta_{\beta\nu} - \delta_{\alpha\nu}\delta_{\beta\mu}$, it follows

$$= \frac{1}{2}R_{ji}R_{ki}\sum_{\alpha,\beta}\sum_{\mu,\nu}\sum_{\gamma} f_{\alpha\beta}\,\varepsilon_{\gamma\alpha\beta}\varepsilon_{\gamma\mu\nu}\hat{\mathbf{e}}_{\mu|ji}\,\hat{\mathbf{e}}_{\nu|ki}\,. \tag{44}$$

f can be identified with the components of solenoidal gyro-vector field $\mathbf{F}$ through

$$\mathbf{F} = (F_x, F_y, F_z) \quad \text{with} \quad F_\alpha = \sum_{\beta=1,\gamma=1}^{3}\varepsilon_{\alpha\beta\gamma} f_{\beta\gamma}\,. \tag{45}$$

Analogously, I can introduce the normal vectors $\boldsymbol{\tau}_{ijk} = \boldsymbol{\tau}_{i,ji,ki}$

$$\boldsymbol{\tau}_{ijk} = (\tau_x, \tau_y, \tau_z)_{ijk} \quad \text{with} \quad \tau_{\alpha|ijk} = \sum_{\beta=1,\gamma=1}^{3}\varepsilon_{\alpha\beta\gamma}\,\hat{\mathbf{e}}_{\beta|ji}\,\hat{\mathbf{e}}_{\gamma|ki} \quad \text{and} \quad \boldsymbol{\tau}_{ijk} = \boldsymbol{\tau}_{i,ji,ki} = \hat{\mathbf{e}}_{ji}\times\hat{\mathbf{e}}_{ki}\,. \tag{46}$$

The index i represents the point of expansion and is replaced by the point $\mathbf{r} \in \mathbb{R}^3$ in the volume element d$\mathbf{r}$. The unit vector field $\boldsymbol{\tau}_{ji,ki}(\mathbf{r}) = \boldsymbol{\tau}_{j-i,k-i}(\mathbf{r})$ can be interpreted as unit vector of direction normal to a surface $\mathcal{S}$. It is the dual vector to the directions vectors $\hat{\mathbf{e}}_{ji}$ and $\hat{\mathbf{e}}_{ki}$. Finally the micromagnetic expression of the scalar spin-chirality field density is the inner product of the gyro-vector field and a normal vector to a surface $\mathcal{S}$ element of a triangle scaled with the area of the triangle element.

$$\chi_{ji,ki}(\mathbf{r}) = \chi_{j-i,k-i}(\mathbf{r}) = \frac{1}{2}R_{ji}R_{ki}\,\mathbf{F}(\mathbf{r})\cdot\boldsymbol{\tau}_{j-i,k-i}(\mathbf{r})\,. \tag{47}$$

Recalling our definition of the topological orbital moment (TOM), $\mathbf{L}_i^{\mathrm{TO}}$, at the ith atomic site given by the sum of the individual orbital moments $\mathbf{L}_{ijk}^{\mathrm{TO}}$:

$$\mathbf{L}_i^{\mathrm{TO}} = \sum_{(jk)}\kappa_{jk}^{\mathrm{TO}}\,\chi_{ijk}\,\boldsymbol{\tau}_{ijk}\,, \tag{48}$$

this will translate to an topological orbital moment density $\ell^{\mathrm{TO}}(\mathbf{r})$ relating the TOM with the gyro-vector field as

$$\ell^{\mathrm{TO}}(\mathbf{r}) = \underline{\underline{\kappa}}^{\mathrm{TO}}\,\mathbf{F}(\mathbf{r})\,, \tag{49}$$

whereas $\underline{\underline{\kappa}}^{\mathrm{TO}}$ is the micromagnetic topological orbital moment tensor given through the microscopic tensor as

$$\underline{\underline{\kappa}}^{\mathrm{TO}} = \frac{1}{2}\sum_{jk} R_{j-i}\kappa^{\mathrm{TO}}_{j-i,k-i}R_{j-i}\,\boldsymbol{\tau}_{j-i,k-i}\otimes\boldsymbol{\tau}_{j-i,k-i}\,. \tag{50}$$

The results are obtained straightforwardly relating all quantities in Supplementary Eq. (48) relative to site i and inserting Supplementary Eq. (47).

Analogously, we can express the chiral-chiral interaction (CCI), given in the lattice model as

$$E^{\mathrm{CC}} = -\frac{1}{2}\sum_{i(jk)}\varkappa^{\mathrm{CC}}_{ijk}\chi^2_{ijk} = -\frac{1}{2}\sum_{i(jk)}\varkappa^{\mathrm{CC}}_{ijk}\left[\mathbf{S}_i\cdot\left(\mathbf{S}_j\times\mathbf{S}_k\right)\right]^2\,, \tag{51}$$

in terms of the micromagnetic model as

$$E^{\mathrm{CC}}_{\mathrm{mm}} = -\frac{1}{2}\sum_{\alpha,\beta=1}^{3}\tilde{\varkappa}^{\mathrm{CC}}_{\alpha\beta}\int \mathrm{d}\mathbf{r}\,F_\alpha(\mathbf{r})F_\beta(\mathbf{r}) = -\frac{1}{2}\int\mathrm{d}\mathbf{r}\,\mathbf{F}(\mathbf{r})\cdot\underline{\underline{\tilde{\varkappa}}}^{\mathrm{CC}}\cdot\mathbf{F}(\mathbf{r})\,, \tag{52}$$

where the sum over the sites i is replaced by the integral over the volume, $\sum_i \rightarrow 1/\Omega\int\mathrm{d}\mathbf{r}$, Ω is the volume per atom, and $\underline{\underline{\tilde{\varkappa}}}^{\mathrm{CC}}$ is the micromagnetic chiral-chiral interaction tensor, whose tensor elements are given by

$$\tilde{\varkappa}^{\mathrm{CC}}_{\alpha\beta} = \frac{1}{4\Omega}\sum_{jk} R^2_{j-i}\kappa^{\mathrm{CC}}_{j-i,k-i}R^2_{k-i}\,\tau_{\alpha|j-i,k-i}\tau_{\beta|j-i,k-i}\,. \tag{53}$$

We express the spin-chiral interaction (SCI), given in the lattice model as

$$E^{\mathrm{SC}} = \sum_i \varkappa^{\mathrm{SC}}_i\mathbf{L}^{\mathrm{TO}}_i\cdot\mathbf{S}_i = \sum_{i(jk)}\varkappa^{\mathrm{SC}}_{ijk}\left(\boldsymbol{\tau}_{ijk}\cdot\mathbf{S}_i\right)\left[\mathbf{S}_i\cdot\left(\mathbf{S}_j\times\mathbf{S}_k\right)\right]\,, \tag{54}$$

in the continuum approximation as

$$E^{\mathrm{SC}}_{\mathrm{mm}} = \sum_{\alpha\beta}\tilde{\varkappa}^{\mathrm{SC}}_{\alpha\beta}\int\mathrm{d}\mathbf{r}\,m_\alpha(\mathbf{r})F_\beta(\mathbf{r}) = \int\mathrm{d}\mathbf{r}\,\mathbf{m}(\mathbf{r})\cdot\underline{\underline{\tilde{\varkappa}}}^{\mathrm{SC}}\cdot\mathbf{F}(\mathbf{r})\,, \tag{55}$$

where $\underline{\underline{\tilde{\varkappa}}}^{\mathrm{SC}}$ is the micromagnetic spin-chiral interactin tensor, whose tensor elements are given by

$$\tilde{\varkappa}^{\mathrm{SC}}_{\alpha\beta} = \frac{1}{2\Omega}\sum_{jk} R_{j-i}\varkappa^{\mathrm{SC}}_{j-i,k-i}R_{k-i}\,\tau_{\alpha|j-i,k-i}\tau_{\beta|j-i,k-i}\,. \tag{56}$$

For cubic crystals all tensors reduce to a diagonal one with one independent constant, e.g. $\underline{\underline{\tilde{\varkappa}}}^{\mathrm{SC}} = \tilde{\varkappa}^{\mathrm{SC}}\underline{\underline{1}}$. Determining the microscopic tensor elements for example by means of density functional calculations, the micromagnetic tensor elements are determined through contractions over the neighboring atoms in dependence on the range of interactions.

Supplementary Note 7 | Phenomenological model

Generalized order parameters. We consider the case of spin magnetic moments with a fixed length $S = 1$. In the general non-collinear case, we define the AFM order parameters $\mathbf{N}_i$ as linear combinations of the local spin moments [12]:

$$\begin{aligned}\mathbf{N}_1 &= (\mathbf{S}_1 - \mathbf{S}_2 + \mathbf{S}_3 - \mathbf{S}_4)/4\,,\\ \mathbf{N}_2 &= (\mathbf{S}_1 - \mathbf{S}_2 - \mathbf{S}_3 + \mathbf{S}_4)/4\,,\\ \mathbf{N}_3 &= (\mathbf{S}_1 + \mathbf{S}_2 - \mathbf{S}_3 - \mathbf{S}_4)/4\,,\end{aligned} \tag{57}$$

which allows us to characterize the magnetic structure with the total spin moment $\mathbf{M} = (\mathbf{S}_1 + \mathbf{S}_2 + \mathbf{S}_3 + \mathbf{S}_4)/4$. In the fully compensated structure with $\mathbf{M} = 0$, Eq. 4 of the main text, the AFM order parameters are mutually orthogonal and obey the relation $\mathbf{N}_1^2 + \mathbf{N}_2^2 + \mathbf{N}_3^2 = 1$ according to

$$\begin{aligned} \mathbf{N}_1 &= (0, \sin\theta \sin\phi, 0)\,, \\ \mathbf{N}_2 &= (\sin\theta\cos\phi, 0, 0)\,, \\ \mathbf{N}_3 &= (0, 0, \cos\theta)\,. \end{aligned} \tag{58}$$

Analogously, we define vectors $\mathbf{C}_i$ as combinations of the (topological) orbital moments $\mathbf{L}_i$ of each sublattice:

$$\begin{aligned} \mathbf{C}_1 &= (\mathbf{L}_1 - \mathbf{L}_2 + \mathbf{L}_3 - \mathbf{L}_4)/4\,, \\ \mathbf{C}_2 &= (\mathbf{L}_1 - \mathbf{L}_2 - \mathbf{L}_3 + \mathbf{L}_4)/4\,, \\ \mathbf{C}_3 &= (\mathbf{L}_1 + \mathbf{L}_2 - \mathbf{L}_3 - \mathbf{L}_4)/4\,, \end{aligned} \tag{59}$$

and the net orbital moment is $\mathbf{L} = (\mathbf{L}_1 + \mathbf{L}_2 + \mathbf{L}_3 + \mathbf{L}_4)/4$. Furthermore, we use the cube diagonals $\mathbf{n}_i$ in the B20 materials to define linear combinations $\mathbf{e}_i$ that point along different Cartesian directions:

$$\begin{aligned} \mathbf{e}_1 &= (\mathbf{n}_1 - \mathbf{n}_2 + \mathbf{n}_3 - \mathbf{n}_4)/4 = \mathbf{e}_y\,, \\ \mathbf{e}_2 &= (\mathbf{n}_1 - \mathbf{n}_2 - \mathbf{n}_3 + \mathbf{n}_4)/4 = \mathbf{e}_x\,, \\ \mathbf{e}_3 &= (\mathbf{n}_1 + \mathbf{n}_2 - \mathbf{n}_3 - \mathbf{n}_4)/4 = \mathbf{e}_z\,. \end{aligned} \tag{60}$$

Symmetry of magnetic phases. Potential magnetic structures can be classified according to the transformation behavior of the components of the order parameters $\mathbf{N}_i$ that form a basis for different irreducible representations of the space group P2$_1$3 of the B20 materials. On the one hand, the most symmetrical among all magnetic structures preserves the cubic symmetry of the paramagnetic phase but breaks time-reversal symmetry owing to finite values $N_{1y} = N_{2x} = N_{3z}$ (all other components vanish) of the order parameters. In this cubic phase the local spin moments point along the cube diagonals, i.e., $\mathbf{S}_i \parallel \mathbf{n}_i$. On the other hand, while the generalized AFM parameters still point along the same directions as in the cubic case, they have different lengths in the orthorhombic phase. If the symmetry is reduced further, we arrive at the triclinic phase where the order parameters $\mathbf{N}_i$ are perpendicular to different Cartesian axes. The latter phase is outside of the scope of the present work.

Magnetic and orbital energy density. To distinguish between cubic and orthorthombic magnetic phases characterized by Supplementary Eq. (58), we analyze the free energy $w = w_{\mathrm{ex}} + w_{\mathrm{orb}}$ of the crystal. Based on symmetry considerations, the magnetic energy density w_{ex} assumes the general form

$$w_{\mathrm{ex}} = \frac{1}{2} J_0 \mathbf{M}^2 + \mathcal{K} \sum_{i=1}^{3} \mathbf{N}_i^4 - \lambda \mathbf{M}\cdot\mathbf{L} + w_{\mathrm{DM}} + w_{\mathrm{ani}}\,, \tag{61}$$

where we introduced phenomenological constants that describe exchange (J_0, $\mathcal{K}$), Dzyaloshinskii-Moriya interaction (w_{DM}), and anisotropy energy (w_{ani}), which we disregard here. The parameter λ controls the interaction between the magnetization and the net orbital moment in uncompensated magnetic structures, which can also be relevant for dynamics or in external magnetic field.

In addition, Supplementary Eq. (61) has to be supplemented by an orbital contribution to the energy density, stemming from the coupling of the magnetic vectors $\mathbf{C}_i$ to the spin texture:

$$w_{\mathrm{orb}} = \sum_{i=1}^{3} \left(\frac{A_{\mathrm{orb}}}{2} \mathbf{C}_i^2 - \mu^{\mathrm{SC}} \mathbf{C}_i \cdot \mathbf{N}_i - \mu^{\mathrm{CC}} \chi_{\mathbf{N}} \mathbf{C}_i \cdot \mathbf{e}_i \right)\,, \tag{62}$$

where we defined $\chi_{\mathbf{N}} = \mathbf{N}_1 \cdot (\mathbf{N}_2 \times \mathbf{N}_3)$, which can be related to the scalar spin chirality χ_{ijk}. These orbital energy terms are mediated by phenomenological constants μ^{SC}, μ^{CC}, and $A_{\mathrm{orb}} > 0$, the latter of which prohibits the formation of the orbital moments in the paramagnetic phase. By minimizing Supplementary Eq. (62) with respect to the orbital moments we find explicit expressions for the magnetic vectors $\mathbf{C}_i$:

$$\mathbf{C}_i = \mu \mathbf{e}_i \chi_{\mathbf{N}} + \frac{\mu^{\mathrm{SC}}}{A_{\mathrm{orb}}} \mathbf{N}_i\,, \tag{63}$$

where $\mu = \mu^{\mathrm{CC}}/A_{\mathrm{orb}}$. This is in agreement with our conclusions drawn from the *ab initio* calculations, where the orbital moment consists of texture-driven and spin-orbital contributions. Substituting the above expression back into Supplementary Eq. (62) leads to the effective orbital energy density $w_{\mathrm{orb}}^{\mathrm{eff}}$ as given by

$$w_{\mathrm{orb}}^{\mathrm{eff}} = -\bar{\kappa}^{\mathrm{CC}} \chi_{\mathbf{N}}^2 - \bar{\kappa}^{\mathrm{SC}} \chi_{\mathbf{N}} (N_{1y} + N_{2x} + N_{3z})\,, \tag{64}$$

where we introduced the phenomenological constants $\bar{\kappa}^{\rm CC} = A_{\rm orb}\mu^2/2$ and $\bar{\kappa}^{\rm SC} = \mu^{\rm SC}\mu$. The first term in Supplementary Eq. (64) can be interpreted as an analog of the CCI favouring the state with maximal chirality, i.e., the cubic phase. The second term constitutes an additional contribution to the anisotropy energy that selects the sign of spin chirality and supports the alignment of the AFM vectors along the cubic axes. In other words, the local orbital moments create via the spin-orbit interaction local "easy" directions for the spins parallel to the cube diagonals $\mathbf{n}_i$. Depending on magnitude and sign of the interaction parameters, various magnetic structures can thus emerge owing to the competition of different spin and orbital energy contributions.

Supplementary References

[1] Kvashnin, Y. O. *et al.* Exchange parameters of strongly correlated materials: Extraction from spin-polarized density functional theory plus dynamical mean-field theory. *Phys. Rev. B* **91**, 125133 (2015).

[2] Liechtenstein A. I., Katsnelson M. I., Antropov V. P. & Gubanov V. A. Local spin density functional approach to the theory of exchange interactions in ferromagnetic metals and alloys. *J. Magn. Magn. Mater.* **67** 65–74 (1987).

[3] Antal, A. *et al.* First-principles calculations of spin interactions and the magnetic ground states of Cr trimers on Au(111). *Phys. Rev. B* **77** 174429 (2008).

[4] Ebert, H. & Mankovsky, S. Anisotropic exchange coupling in diluted magnetic semiconductors: Ab initio spin-density functional theory. *Phys. Rev. B* **79**, 045209 (2009).

[5] Moriya, T. New Mechanism of Anisotropic Superexchange Interaction. *Phys. Rev. Lett.* **4**, 228–230 (1960).

[6] Dzyaloshinskii I. E. Thermodynamic theory of "weak" ferromagnetism of antiferromagnetics. *J. Phys. Chem. Solids* **4**, 241 (1958).

[7] Hoffmann, M. & Blügel, S. Systematic derivation of realistic spin-models for beyond-Heisenberg solids from microscopic model. Preprint at arXiv:1803.01315 (2019).

[8] Brinker, S., dos Santos Dias, M. & Lounis, S. The chiral biquadratic pair interaction. *New J. Phys.* **21**, 083015 (2019).

[9] Lászlóffy, A., Rósza, L., Palotás, K., Udvardi, L. & Szunzogh, L. Magnetic structure of monatomic Fe chains on Re(0001): emergence of chiral multi-spin interactions. *Phys. Rev. B* **99**, 184430 (2019).

[10] Akulov, N. S. Über das magnetische Quadrupolmoment des Eisenatoms. *Z. Phys.* **57**, 249 (1929); ibid. **69**, 78 (1931).

[11] Blügel, S. Magnetische Anisotropie und Magnetostriktion, Schriften des Forschungszentrums Jülich ISBN 3-89336-235-5 (1999).

[12] Gomonay, E. V. & Loktev, V. M. Spintronics of antiferromagnetic systems (Review Article). *Low Temp. Phys.* **40**, 17 (2014).
